# Supplementary material for: Unmasking cellular response of a bloom-forming alga to viral infection by resolving expression profiles at a single-cell level
Source: PLoS Pathog. 2019 Apr 24;15(4):e1007708. doi: 10.1371/journal.ppat.1007708 (PMC6502432; doi:10.1371/journal.ppat.1007708)
Supplement: S1 Text — (DOCX) [file ppat.1007708.s004.docx]

**S1 Text**


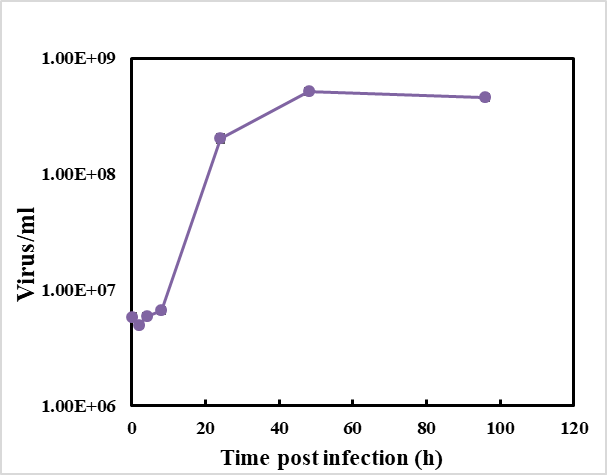

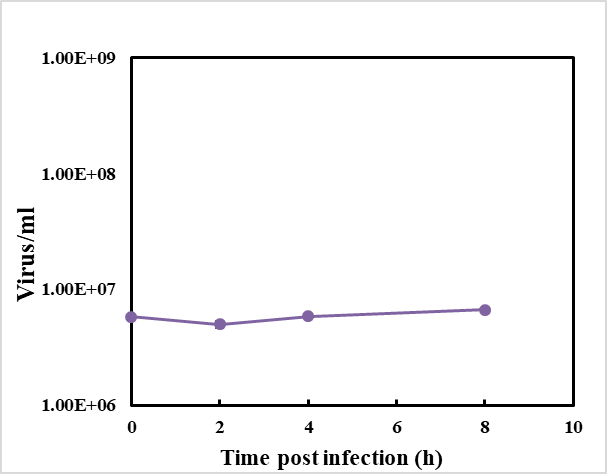


**Figure A: Extracellular viral production during EhV infection of *E. huxleyi* cells.** Abundance of extracellular viruses (EhV) were monitored using flow-cytometry as described in Materials and Methods. Inset highlighted there is no viral production within 8 hpi**.**

**Figure B: Expression level of viral genes during mock infection with UV- inactived EhV virions.** Expression level presented as Et (30-Ct) values of selected viral genes in *E. huxleyi* 2090 culture infected with EhV 201 and UV-inactived EhV 201 at 4 hpi. Viral genes were selected based on their temporal expression during infection. EPVG_00014 and EPVG_00014 are expressed at early phase and AET97971.1 and EPVG_00010 are late genes.

**Figure C: Fraction of infectious particles during EhV infection.** The percentage of infection particles in the culture medium during EhV infection of *E. huxleyi* cells was measure using the MPN method as described in Materials and Methods.
